# An *Oenothera biennis* Cell Cultures Extract Endowed with Skin Anti-Ageing Activity Improves Cell Mechanical Properties

**DOI:** 10.3390/metabo11080527

**Published:** 2021-08-09

**Authors:** Sara Ceccacci, Adriana De Lucia, Annalisa Tito, Assunta Tortora, Danila Falanga, Stefania Arciello, Giovanni Ausanio, Chiara Di Cicco, Maria Chiara Monti, Fabio Apone

**Affiliations:** 1 Department of Pharmacy, University of Salerno, 84084 Fisciano, Italy; sceccacci@unisa.it; 2 PhD Program in Drug Discovery and Development, Department of Pharmacy, University of Salerno, 84084 Fisciano, Italy; 3 Arterra Bioscience SpA, 80142 Naples, Italy; adriana@arterrabio.it (A.D.L.); annalisa@arterrabio.it (A.T.); assunta@arterrabio.it (A.T.); danila@arterrabio.it (D.F.); stefania@arterrabio.it (S.A.); fapone@arterrabio.it (F.A.); 4 CNR-SPIN Department of Physics, University of Naples “Federico II”, 80125 Naples, Italy; ausanio@unina.it; 5 Department of Neuroscience and Reproductive and Odontostomatological Sciences, University of Naples “Federico II”, 80131 Naples, Italy; chiara.dicicco92@gmail.com; 6 Vitalab Srl, 80142 Naples, Italy

**Keywords:** metabolomics, molecular networks, hydrophilic extract of *Oenothera biennis* cell cultures, matrix collagen contraction, skin aging

## Abstract

Skin aging is a very well-known process setting a gradual worsening of skin mechanical features due to a decline in the production of the extra-cellular matrix machinery and to a concurrent change in the contraction process. To slow this progression, it is crucial to induce the expression of several proteins able to promote elastic fibers formation and tissue repair. Here, the *Oenothera biennis* cell culture aqueous extract has been investigated from a chemical point of view and then it was tested in vitro, in cell, and in ex vivo experiments as adjuvant in counteracting skin aging. Accordingly, it has been shown that the *Oenothera biennis* extract was able, by increasing MYLK gene expression, to promote matrix collagen contraction, actin polymerization, and the production of essential ECM proteins.

## 1. Introduction

Skin aging is a very well-known process induced by endogenous and exogenous factors. During senescence, all the cutaneous physiological functions inexorably degenerate, and this progressive deterioration damages the skin [1]. Indeed, the skin gradually drops its mechanical features, owed both to a decline in the production of the extra-cellular matrix proteins and to the concurrent change in the contraction process [2]. The most important dermis extra-cellular matrix components are proteins such as collagen I and III, laminin, periostin, tenascin, elastin, fibronectin, and proteoglycans, as their relative amount and folding state play a key role in the interaction between the cells and the matrix, guaranteeing the proper texture of the dermis [3]. For instance, in the extra cellular space, fibronectin plays a key role in matrix assembly as it forms a bridge between cell surface receptors (e.g., integrins) and collagen, proteoglycans, and other focal adhesion molecules. Laminins contribute to the structure of the extracellular matrix and modulate adhesion, differentiation, migration, stability of phenotype, and resistance towards apoptosis. Elastin is also important for cell adhesion and cell migration, and it has the ability to participate in cell signaling. Fibrillins similarly interact closely with tropoelastin and integrins, and they are important for the assembly of elastins into elastic fibers. Fibulins are tightly connected with basement membranes, elastic fibers, and other components of extracellular matrix, and participate in elastic fibers formation. Tenascins are extracellular matrix (ECM) polymorphic glycoproteins which mediate fibrotic processes to enable effective tissue repair [4]. Additionally, the skin contractile process is also based on the action of fibroblasts, the most abundant cells in the dermal matrix; they set up the proper tension of the skin, promoting the contacts between the matrix components, which are in turn responsible for the dermis compactness, density and resistance. At the basis of their cellular cytoskeleton organization, there is the actin-myosin machinery, which determines potential changes in their shape and in their structure. Indeed, the binding of actin and myosin induces a more compact cellular conformation, bringing the fibers closer together, guaranteeing the development of a healthy and compact tissue and preventing fiber disintegration resulting in wrinkles. However, fibroblast ability to contract and stretch is partially lost along years because the aged cells are no longer able to act correctly and completely. Due to a lower mechanical force, fibroblasts go to a more rounded and distorted morphology from the elongated one [5]. Some evidence has indicated that, during aging, the synthesis of a protein called MYLK (MYosin Light chain Kinase) decreased significantly. MYLK possesses a kinase activity exerted by the phosphorylation of a specific myosin domain, named the myosin regulatory light chain, able to induce actin binding and therefore promote cell contractility [6]. When a low activity of this protein was detected, due, for instance, to a lesser protein expression, a corresponding reduction of matrix collagen contraction [7] and actin polymerization [8] were measured. Actin cytoskeleton assembly is linked not only to the cell movement and ability to contract, but also to the production of ECM matrix protein through the activation of TGFβ-II receptor (TGFBRII) [9]. TGFBRII belongs to the TGF-β cell surface receptor complex that, through activation of Smad transcription factors, regulates the expression of many genes encoding components of ECM, including collagen, laminins, fibronectin and proteoglycan [10]. Actin cytoskeleton disassembly downregulates TGFβ-II receptor. This down-regulation in turn decreases the production of collagen and other ECM proteins, resulting in a loss of dermal mass and skin fragility.

Actually, many cosmetic ingredients aim at stimulating the synthesis of several key factors responsible for maintaining the correct compactness of dermal matrix and a good skin contraction. In this scenario, the extracts derived from the species *Oenothera biennis* (Evening Primrose), belonging to the family Onagraceae, are of a wide interest due to their content of bioactive compounds such as fatty acids, phenols, triterpens and flavonoids, which have already been tested in the treatment of various skin pathological diseases [11]. Some of these metabolites, in turn, are reported to suppress inflammation mediators such as interleukin 1β (IL-1), interleukin 6 (IL-6), cytokines and tumor necrosis factor α (TNF-α) [12]. Moreover, extracts of *Oenothera biennis* aerial parts protected HaCaT cells from H_2_O_2_-induced DNA damage and cell death by blocking cellular damage due to oxidative stress through a mechanism that involved ROS elimination and Nrf2/HO-1 signaling pathway [13]. Furthermore, *Oenothera biennis* oil, which is rich in lipids, has been proposed as a good moisturizer for eczema patients thanks to its capacity to easily penetrate the skin [14]. Regrettably, extract preparations of cultivated plants, engaged in cosmetics, may include some disadvantages: first, they can be contaminated by toxic or allergenic substances such as pesticides, fertilizers or pollutants; then, the plants are subject to unpredictable stress and seasonal conditions or variations in nutrient availability, which can induce the synthesis of unexpected metabolites. Additionally, the extracts may contain pathogenic microorganisms, which reduce the quality of the final products. All these drawbacks, in turn, converge into the risk of having a variable content of secondary metabolites and low reproducibility. To overcome these weaknesses, the use of plant cell cultures in cosmetics is becoming more popular and very much appreciated, since it overcomes many of the above mentioned disadvantages [15]. 

In this study, a hydrophilic extract of *Oenothera biennis* cell cultures (ObHEx) has been fully characterized by advanced mass spectrometric-based approaches, assisted by bioinformatics and tested in multiple biochemical and biological assays. The mixture contains bioactive compounds of mainly lignans and triterpenes, such as arjunolic and asiatic acid, which have been previously associated to pro-collagen I production in human fibroblasts [16,17]. The extract was investigated for its capacity to increase the expression of MYLK, to promote matrix collagen contraction, actin polymerization and the production of TGFβ-regulated ECM proteins, which favour the contraction of the dermis, thus slowing down skin aging.

## 2. Results

### 2.1. Qualitative and Quantitative Analysis of the Water-Soluble Extract of Oenothera Biennis Cell Culture

**Figure 1:**
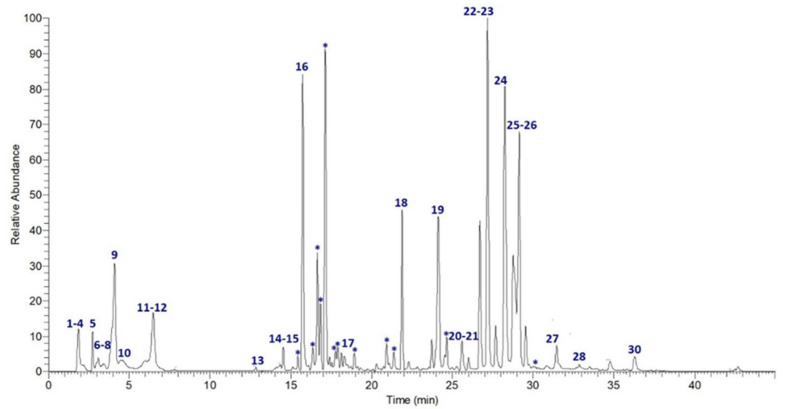
Extracted-ion chromatogram of the main metabolites identified in ObHEx. Those indicated by * were included in molecular networks.

UPLC-MS/MS analysis of ObHEx was performed and high-resolution spectrometric data were exploited for the chemical characterization using Global Natural Products Social Molecular Networking (GNPS), a web-based mass spectrometry system that aids in the identification and annotation of natural products (NPs) [18]. It aims at being an open-access knowledge base for community-wide organizations and for the sharing of raw, processed, or annotated fragmentation mass spectrometry data (MS/MS). Specifically, a GNPS spectral library search and a Feature-Based Molecular Networking (FBMN) job were performed: the first analysis allowed us to identify natural compounds, comparing their MS/MS spectra with those of structurally characterized metabolites, the second one to group related NPs within a network since similar MS/MS fragmentation patterns were exhibited by structurally similar molecules [19]. As shown in Figure 1 and Table 1, many NPs were undoubtedly identified as belonging to several classes of secondary metabolites, mainly to lignans (salvadoraside and liriodendrin) and triterpenes (myrianthic acid, arjunolic acid, asiatic acid, and hederagenin). Chemical species not identified by GNPS were assigned accordingly to literature.

**Figure 2:**
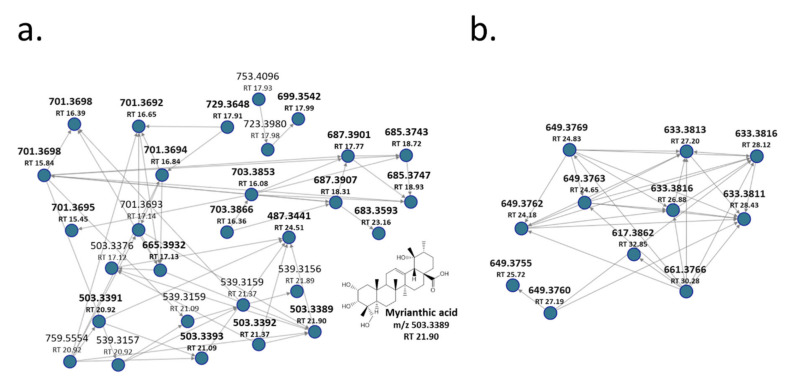
(**a**,**b**) Molecular networks (MNs) showing the presence of other uncharacterized triterpenes in ObHEx.

As an example of the use of GNPS, the network derived from FBMN analysis has been reported in Figure 2a, showing that ObHEx contained many other uncharacterized triterpenes related to myrianthic acid (**18**, *m*/*z* 503.3389, RT 21.89 min: myrianthic acid identification has been confirmed through a comparison with its analytical standard). For instance, various previously uncharacterized species at *m/z* 701 and 665 were identified as glycosylated forms related to myrianthic acid or to its isomers, respectively hydrated or not with two molecules of H_2_O.

Furthermore, reported ions at *m/z* 729, 703, 699, 687, 685, 683 derived respectively from the glycosylation plus two molecules of H_2_O of species at *m/z* 531, 505, 501, 489, 487, and 485: their univocal identification has not been achieved, but they all are supposed to be triterpenes, being related to myrianthic acid or to its congeners, due to their MSMS fragmentation pathway. Recently, many triterpenes with *m/z* 501 and 485 were isolated from another species of Oenothera, *Oenothera maritima*, and their structure has been elucidated on the basis of spectroscopic data, thus well correlating with our results [20].

FBMN analysis also provided information on the metabolites at *m/z* 617.3862 (MS^2^ ions at *m/z* 453, 145 and 119) present in molecular networks shown in Figure 2b and not reported in literature for Oenothera species. This *m/z* value and its fragmentation match with 2-O-E-p-coumaroyl alphitolic acid or its isomers, proving, at least, the presence of triterpene coumaroyl and also caffeoyl esters in the extract. Indeed, MS^2^ spectra of species at *m/z* 649 (RT 25.72 and 27.19) showed the presence of ions at *m/z* 179, 161, and 135 due to the cleavage of caffeic acid moiety, whereas the MS^2^ spectra of the other species reported in Figure 2b at *m/z* 633 (RT 26.88, 27.20, 28.12 and 28.43), 649 (RT 24.18, 24.65, 24.83), and 661 (RT 30.28) showed ions at *m/z* 145 and 119, which were characteristic for coumaroyl moiety.

After, the content of some identified lignans and triterpenes was determined. Quantification methods were validated as reported in Table 2. All calibration curves showed good linearity (R^2^ ≥ 0.9911) within the tested ranges. Moreover, the limit of detection (LOD) and the limit of quantification (LOQ) indicated that the used methods were distinguished by high sensitivity. The obtained results from the quantitative analysis (Table 3) showed that liriodendrin and hederagenin were the main represented lignan and triterpene, respectively.

### 2.2. Cytotoxicity Tests (MTT Assay)

Cytotoxicity tests were performed on Human Dermal Fibroblasts (HDF) to determine the range of concentrations in which ObHEx was not dangerous for the human cells in the growth phase. The treatment was carried out with increasing concentrations of the extract between 0.05% and 0.0004% (500 μg/mL and 4 μg/mL) for approximately 48 h, and, after a period of 16 h incubation, the colorimetric reaction was measured at 595 nm. The results of the MTT indicated that the hydro-extract concentrations equal to or less than 0.01% did not cause any toxicity in the cells. The CC_50_ was 0.026% as calculated using the software provided on the website https://www.aatbio.com/tools/ic50-calculator (accessed on 29 December 2020). For convenience, we decided to use the concentration of 0.006% as the maximum dose in the cell assays.

### 2.3. Analysis of MYLK Gene Expression in HDF

**Figure 3:**
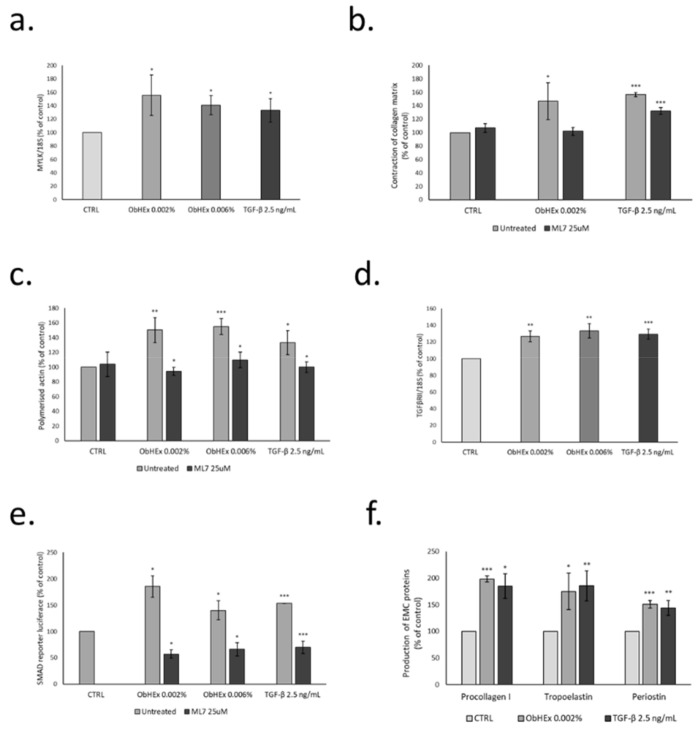
Bar graphs showing the ability of ObHEx (**a**) to increase the expression of the MYLK gene, (**b**) to improve contractile capacity of collagen fibers, (**c**) to stimulate actin polymerization, (**d**,**e**) to activate TGF-β signaling and (**f**) to boost the production of pro-collagen I, tropoelastin, and periostin in HDF. The bars represent the standard deviations while asterisks indicate significant variations (* *p* < 0.05; ** *p* < 0.01; *** *p* < 0.001), according to Student’s *t*-test.

The activity of ObHEx was tested in HDF on the expression of the MYLK gene, which encodes for the kinase responsible for the light chain phosphorylation of myosin (Figure 3a). The results indicated that the treatment with the extract at both concentrations increased MYLK gene expression by about 50%, similar to the positive control TGF-β.

### 2.4. Analysis of the Contraction Capacity of a Collagen Matrix

To evaluate the activity of ObHEx on the contractile capacity of collagen fibers, we used HDF dispersed in collagen gel discs [21]. As shown in Figure 3b, the extract, at the lower concentration, induced a significant increase in the contraction of the collagen disc, suggesting a potential effect on collagen firmness. The treatment with ML7 (1-(5-iodonaphthalene-1-sulfonyl)-1H-hexahydro-1,4-diazepine), a MYLK inhibitor, abolished this increase, indicating that both the extract and TGF-β act through MYLK for collagen disk contraction.

### 2.5. Measure of Actin Polymerization Level

The capacity of ObHEx to stimulate actin polymerization was investigated by measuring the level of polymerized actin in the cells, treated with the extract and the positive control TGF-β, alone and in the presence of ML7. As shown in Figure 3c, the treatment with both the concentrations of the extracts yielded an increase in the amount of polymer actin by about 50%, compared to 33% of TGF-β. Again, the pre-incubation with ML7 completely abolished this effect, suggesting an involvement of MYLK in the polymerization of actin filaments.

### 2.6. Analysis of TGFβRII/SMAD Pathway in HDF

To verify whether the increase in actin polymerization and collagen contraction of cells treated with ObHEx was also associated with an activation of signal transduction pathway mediated by TGFβRII, we firstly observed the expression of TGFβRII gene and then transfected HDF with the SMAD2-luciferase reporter plasmid and evaluated the increase in luciferase activity in response to ObHEx treatment. The results, reported in Figure 3d,e, indicated that the treatment with ObHEx at 0.002% and 0.006% increased the expression of TGFβRII in a way similar to the TGF-β used as positive control and, parallelly, the luciferase activity linked to SMAD was increased by 80 and 40%, respectively. A significant signal reduction was obtained when the cells were treated with ML7, showing also that this activity was linked to MYLK.

### 2.7. Analysis of Pro-Collagen I, Tropoelastin and Periostin

As a consequence of TGFβRII signal transduction activation, we analyzed the synthesis of the main extracellular matrix proteins such as procollagen type I, tropoelastin and periostin in HDF treated with ObHEx at 0.002%. As shown in Figure 3f, ObHEx increased the production of the indicated proteins by about 98%, 75%, and 51%, respectively, similar to the positive control TGF-β. The measures were performed by ELISA assay, using specific antibodies against pro-collagen I, tropoelastin, and periostin.

### 2.8. Analysis of MYLK, Phospho-Myosin, Collagen I and Tropoelastin on Ex-Vivo Skin Explants

**Figure 4:**
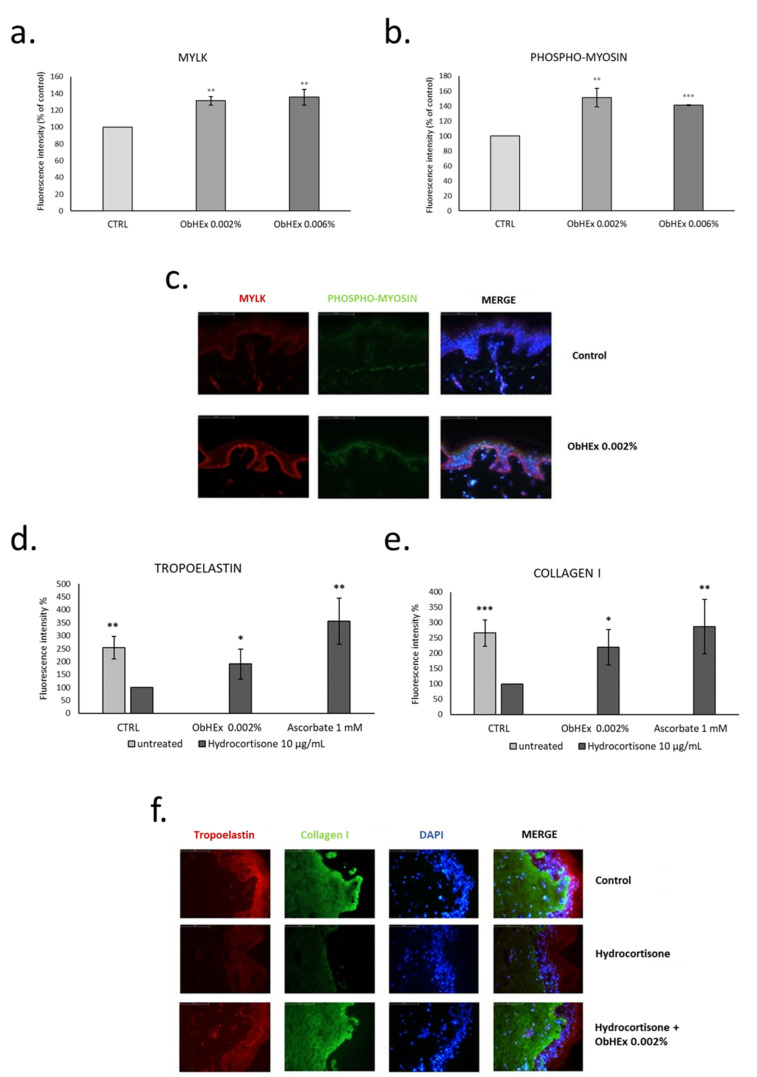
Bar graphs showing the effect of ObHEx to increase the production of MYLK (**a**) and phospho-myosin (**b**) in skin explants and its ability to restore the amount of tropoelastin (**d**) and collagen I (**e**) after treatment with hydrocortisone; the quantization was performed by detection with antibody labeled with fluorophore. The bars represent the standard deviations while asterisks indicate significant variations (* *p* < 0.05; ** *p* < 0.01; *** *p* < 0.001) according to Student’s *t*-test. (**c**,**f**) Photographs of skin sections in which the indicated proteins have been labeled with specific antibody both before and after treatment with the extracts. The abbreviation “MERGE” indicates the overlapping of the photographs where the fluorescence of the specific protein is highlighted with those in which the nuclei have been stained with 4’,6-diamidine-2-phenylindole (Dapi).

As shown in Figure 4a–c, ObHEx produced a significant increase in the production of MYLK and phosphorylated myosin in human skin explants. 

Collagen I and tropoelastin induction were also analyzed in skin explants pretreated with ObHEx and then treated with hydrocortisone for 8 days to simulate chronological ageing [22]. The treatment with hydrocortisone reduced the amount of collagen I and tropoelastin by more than 100%, and the presence of 0.002% ObHEx restored the amount of tropoelastin by almost 91% and of collagen I by 120%, compared to the ascorbate used as positive control (Figure 4d–f). This suggests a potential anti-aging effect of ObHEx, particularly effective in increasing the firmness of the skin by acting on the dermal matrix components. 

### 2.9. Atomic Force Microscopy (AFM) in Skin Explants

**Figure 5:**
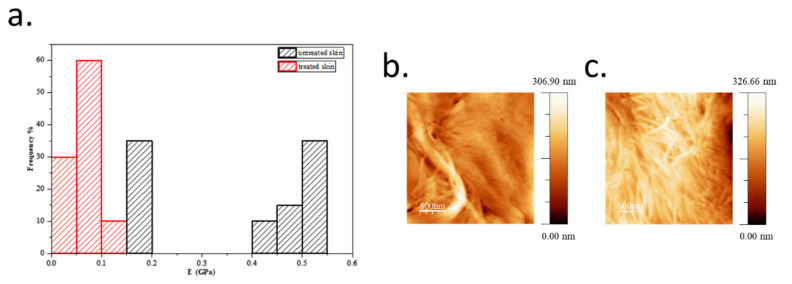
(**a**) The histogram shows the distribution of Young’s modulus values calculated applying Hertz’s model on Force curves in ten different points of the same untreated (black bars) and treated (red bars) skin sample surfaces. (**b**,**c**) Images of fibrils in the case of untreated (**b**) and treated (**c**), which were acquired in contact mode.

In order to collect more information about the skin biomechanical properties promoted by the treatment with ObHEx, we evaluated elasticity, an intrinsic mechanical property of skin samples in terms of their Young’s moduli [23]. As described in Section 4, to calculate the elastic modulus, both on treated and untreated skin samples, we collected Force curves with an Atomic Force Microscope (AFM) and fit them with Hertz model [24,25]. As shown in Figure 5, the treatment of the skin samples with the extract had a lower Young’s module values compared to untreated samples, suggesting an improvement of skin elastic properties. In particular, untreated skin samples disclosed a mean Young’s modulus value of 0.37 ± 0.15 GPa, while skin samples treated with ObHEx showed a lower mean value of 0.07 ± 0.02 GPa. The Young’s modulus values of the skin samples were in accordance with literature [26,27,28]. 

## 3. Discussion

Plant cell cultures constitute the most promising approach for a sustainable production of plant secondary metabolites of commercial interest, offering a continuous supply of material by means of large-scale culture and constituting a sustainable and eco-friendly system [29,30]. Extracts from plant cell cultures have found promising applications in the healthcare and cosmetic sectors, as they present some relevant advantages: (i) they contain metabolites bio-synthesized in controlled growth laboratory conditions; (ii) they derive from standardized production processes, guaranteeing the same qualitative and quantitative characteristics of the obtained species; (iii) the extracts are free of contaminants such as microorganisms, herbicides, pesticides and fungicides; (iv) they are independent from geographical or environmental fluctuations and the plant species can be conserved for future generations. In this contest, an *Oenothera biennis* cell culture aqueous extract (ObHEx) was examined as a promising source of bioactive molecules endowed with skin anti-aging activity. Indeed, ObHEx has been investigated by high resolution UPLC-MSMS analyses coupled to bioinformatics approaches to achieve a wide structural characterization. Compound characterization has been realized mainly by using GNPS to speed up the identification process, compare the MS/MS spectra with those of structurally characterized metabolites, and, furthermore, to disclose new unexpected species, grouping similar NPs within a network. Moreover, retention time, accurate mass measurements and MS^2^ analyses were also compared with those of standards, and all data were matched with literature. Bioactive lignans and triterpenes, such as salvadoraside, liriodendrin, myrianthic acid, arjunolic acid, asiatic acid, and hederagenin were identified. Liriodendrin [31] and myrianthic acid [32] exhibited strong antioxidant activity, while hederagenin showed skin anti-aging properties due to a reduction of cellular oxidation and the activation of proteasome function [33]. However, arjunolic and asiatic acids were considered mainly responsible for some of the following tested activities, since they stimulate collagen I synthesis. Indeed, it has been demonstrated that ObHEx improved the skin biomechanical properties, which mostly depend on the relative amount of the different components of the ECM and on how the fibroblasts are capable of contracting and providing the right tension to the dermal fibers. In fact, ObHEx promotes collagen matrix contraction and actin polymerization by increasing the expression of MYLK gene, enhancing the contraction force of dermal fibroblasts. Assembly of actin cytoskeletron upregulates TGF-β type II receptor and, consistent with the stimulation of TGF-β/Smad signaling, increases the levels of TGF-β regulated ECM proteins. Indeed, ObHEx induces the production of type I collagen, periostin, and tropoelastin. Therefore, all these properties make it a good promising candidate ingredient to be used in cosmetic formulations to fight the age-associated loss of skin firmness and elasticity. This assumption was supported by the results obtained in the AFM analysis, which showed that the treatment of skin slices with ObHEx produced an improvement of the skin mechanical properties, detected as a decrease in the Young’s modulus, indicating a significant reduction in skin stiffness and rigidity.

## 4. Materials and Methods

### 4.1. Plant Tissue Cultures and Extract Preparation

*Oenothera biennis* plants were provided by GEEL Floricultura s.s and were of Italian origin (avoiding the application of Nagoya protocol). Cell culture were obtained from leaves of *Oenothera biennis* plants by inducing the proliferation of meristematic cells on solid agar plates until obtaining calluses. The cells were transferred to the liquid growth medium (Gamborg B5, supplemented with 2,4 dichlorophenoxyacetic acid (1 mg/L), adenine (1 mg/L), and kinetine (0.01 mg/L)). Then, the cells were grown as suspension cultures under orbital shaking. Once the cultures of about 150 g/L were obtained, the cells were collected and lysed in a phosphate buffer (PBS) at pH 7.4 to prepare a water-soluble extract, which was lyophilized. The powder was dissolved in water or cell culture media at the appropriate concentrations for testing. 

### 4.2. UPLC-MS/MS Analysis for Chemical Characterization

ObHEx (50 mg/mL) was prepared and submitted to a biphasic butanol/water extraction. The butanolic fraction was dried and dissolved in methanol (10 mg/mL) before the UPLC-MS/MS analysis. It was carried out on a Q-Exactive Classic Mass Spectrometer from Thermo-Scientific (Waltham, MA, USA) equipped with a Thermo Scientific™ UltiMate™ 3000 UPLC system. All the chromatographic runs were performed using a Phenomenex Luna^®^ C18 100 Å (150 × 2.0 mm, particle size 3 µm) column at 40 °C and flow rate of 0.200 mL/min. The injection volume was 5 µL. The mobile phase consisted of A (water from ROMIL Ltd, Convent Drive, Waterbeach, Cambridge, UK at 0.1% acetic acid) and B (100% acetonitrile from ROMIL Ltd, Convent Drive, Waterbeach, Cambridge, UK) using a gradient elution of 5% B at 0–5 min, 5–14% B at 5–8 min, 14–32% B at 8–11 min, 32–95% B at 11–32 min, 95–98% B at 32–33 min, 98% B at 33–38 min, 98–5% B at 38–39 min, and 5% B at 39–45 min. All the MS and MSMS analyses were carried out in ESI negative mode with the sheath gas flow rate at 30 (arbitrary units), the auxiliary gas flow rate at 5 (arbitrary units), the spray voltage at 3.2 kV, and the capillary temperature and the auxiliary gas heater temperature at 300 °C. Data were acquired with a Full MS/dd-MS2 (Top5) mode. Full MS settings were: resolution of 70.000, AGC target of 1 × 10^6^, maximum IT of 200 ms and scan range from 100 to 800 *m/z*. dd-MS2 settings were: resolution of 17.500, AGC target of 2 × 10^5^, maximum IT of 65 ms, isolation window of 1.5 *m/z* and NCE of 35.

### 4.3. Global Natural Products Social Molecular Networking Analysis

For metabolite identification, Global Natural Products Social Molecular Networking (GNPS at https://gnps.ucsd.edu, accessed on 29 December 2020) was used. All those MS and MSMS signals not assigned by GNPS were carefully inspected and assigned accordingly to literature. Raw files were converted to mzXML format by MS Converter General User Interface software (ProteoWizard 3.0; http://proteowizard.sourceforge.net/project.shtml, accessed on 29 December 2020) before the GNPS spectral library search analysis. It was achieved using precursor ion mass tolerance of 0.025 Da, fragment ion mass tolerance of 0.02 Da, minimum matched peaks of 2 and score threshold of 0.7. The results obtained were manually verified. mzXML data were processed using Mzmine 2.53 before the Feature-Based Molecular Networking (FBMN) job on GNPS. The mass detection step was performed using the centroid mass detector while keeping the noise level at 5 × 10^3^. The Automated Data Analysis Pipeline (ADAP) chromatogram building was realized with the following settings: min group size in number of scans of 5, group intensity threshold of 5 × 10^3^, min highest intensity of 5 × 10^4^, *m/z* tolerance of 0.01 *m/z* or 10 ppm. The chromatogram deconvolution was achieved using Wavelets (ADAP) as an algorithm, S/N threshold of 3, min feature height of 1 × 10^5^, coefficient/area threshold of 5, peak duration range of 0.10–3.00 min and RT wavelet range 0.00–0.05. Chromatograms were deisotoped using the isotopic peaks grouper algorithm with a *m/z* tolerance of 0.001 *m/z* or 5.0 ppm and a RT tolerance of 0.10 min. FBMN job was performed using parent mass tolerance of 0.02 Da and a MS^2^ fragment ion tolerance of 0.02 Da. Edges were filtered to have a score threshold of 0.7 and minimum 2 matched peaks. Moreover, the maximum number of neighbor nodes for each node was set to 10.

### 4.4. Quantitative Analysis of Lignans and Triterpenes

The same UPLC conditions reported for the qualitative analysis were used for the quantitative analysis of lignans, while for that of triterpenes, they were optimized in order to separate two pairs of isomers, arjunolic and asiatic acid. The analysis was performed on a Q-Exactive Classic Mass Spectrometer as previously described. The separation was carried out by a Phenomenex Kinetex^®^ EVO C18 300 Å (150 × 2.1 mm, particle size 5 µm). The mobile phase consisted of A (5 mM ammonium acetate aqueous solution, pH 9.00 adjusted by ammonium hydroxide) and B (100% acetonitrile) using a gradient elution of 17–28% B at 0–18 min, 28–65% B at 18–22 min, 65–75% B at 22–26min, 75–95% at 26–26, 5 min, 95% at 26,5–30 min, 95–17% at 30–30,1 min, 17% at 30,1–42 min. The flow rate was 0.450 mL/min and the injection volume was 5 μL. For both lignans and triterpenes, data were acquired with a Full MS-SIM and PRM mode. Full MS-SIM settings were: resolution of 70.000, AGC target of 3 × 10^6^, maximum IT of 200 ms and scan range from 200 to 800 *m/z* for lignans and from 400 to 850 *m/z* for triterpenes. PRM settings were: resolution of 70.000, AGC target of 2 × 10^5^, maximum IT of 100 ms, isolation window of 1.0 *m/z* and NCE of 35 in the case of lignans and 50 in that of triterpenes. We purchased salvadoraside (#598- XS172930) from Biosynth Carbosynth (Staad, St. Gallen, Switzerland), liriodendrin (#SMB00181) and arjunolic acid (#SMB00119) from Sigma–Aldrich (St. Louis, MO, USA), asiatic acid (#0027) from Extrasynthèse (Genay, Lyon, France) and hederagenin (#89706) from PhytoLab GmbH & Co.KG (Vestenbergsgreuth, Bayern–Mittelfranken, Germany). Myrianthic acid was a gift of Prof. Maria Valeria D’Auria, University of Naples. The calibration curves were obtained by injecting standards solutions in the concentration range of 0.25–25 µM for lignans and 0.1–25 µM for triterpenes. Both the pure salvadoraside and liriodendrin showed two LC-MSMS peaks, probably due to chemical equilibrium established in the solution. The limit of detection (LOD) and limit of quantification (LOQ) for standards were determined on the basis of the signal to noise (S/N) ratio. 

### 4.5. Skin Cell Cultures and Explants

Human dermal fibroblasts (HDF) were maintained in Dulbecco’s Modified Eagle Medium (DMEM; Sigma–Aldrich, St. Louis, MO, USA) supplemented with 10% of fetal bovine serum (FBS; Sigma–Aldrich, St. Louis, MO, USA) in 95% air, 5% CO_2_, and humidified atmosphere at 37 °C. Skin explants, obtained from the skin of healthy female donors (aged 44–47) at the surgery center Villa Cinzia (Naples, Italy), were cultured in 24-transwell plates in DMEM/FBS plus antibiotics in air–liquid conditions at 37 °C in 5% CO_2_ humidified air. All donors had given their written informed consent for the use of the skin tissues, according to the Declaration of Helsinki.

### 4.6. Cytotoxicity Test

Cytotoxicity tests were based on the use of the MTT compound [3-(4,5-dimethylthiazolyl)-2,5-diphenyltetrazolium-bromide] [34]. The cells were grown in 96-well plates in the DMEM (Dulbecco’s Modified Eagle Medium) culture medium, supplemented with 10% fetal bovine serum, for approximately 8 h. After treatment with ObHEx between 0.05% and 0.0004% (500 μg/mL and 4 μg/mL) for 48 h, the cells were washed in PBS and incubated with 100 μL/well of “reaction buffer” containing: 10 mM of Hepes, 1.3 mM CaCl_2_, 1 mM MgSO_4_, 5 mM of glucose, and 0.5 mg/mL of MTT colorimetric substrate in buffer PBS at pH 7.4. After 3 h of incubation at 37 °C in 5% CO_2_, 100 μL of solubilizing solution containing 10% of Triton-X100, 0.1 N of HCl in absolute isopropanol were added to each well. After 16 h incubation, the colorimetric reaction was measured at 595 nm with the Victor3 plate reader (PerkinElmer, Waltham, MA, USA). 

### 4.7. Analysis of the Expression of the MYLK and TGFβRII Gene in HDF

Per well, 1 × 10^5^ of HDF were grown in 6-well plates in DMEM at 2% FBS, after 24 h FBS was further diluted to 0.5% and the cells were treated with 0.002% and 0.006% concentrations of ObHEx and TGF-β 2.5 ng/mL, followed by an incubation of 2 h for MYLK and 48 for TGFβRII. For RNA extraction, the “GenElute™ Total RNA Purification” kit purchased by the company Merck (Darmstadt, Germany) was used. After the indicated treatments, the cells were washed with PBS, collected in lysis buffer and subjected to the extraction procedure as reported. The samples were treated with DNase I (Ambion, Austin, TX, USA) at 37 °C for 30 min to remove genomic DNA contaminant. From each sample, 2 µl were loaded onto gel 1% agarose in the presence of denaturing loading dye for the purpose of quantizing the amount of RNA in reference to a specific marker for RNA (ThermoScientific, Waltham, MA, USA). GeneTools (PerkinElmer, Waltham, MA, USA) has been used as a software for quantization. Next, 300 ng of total RNA was retro-transcribed using the enzyme reverse transcriptase (ThermoScientific, Waltham, MA, USA). Semi-quantitative RT-PCR were conducted using as internal standards the pair of universal primers 18S primer/competimer (Ambion, Austin, TX, USA). The PCR products were separated on 1.5% agarose gel, viewed using the Geliance tool (PerkinElmer, Waltham, MA, USA) and analyzed by densitometry using the Genetools software. The sequences of the primers used for amplification were the following: MYLK FW: ATCAAACTGTCAAGTTCAG, MYLK Rv: AGGCACTGCGTGCAGTCCA, TGFBR2 FW: GTCACTGACAACAACGGT, TGFBR2 RV: ATGTCAGAGCGGTCATCT.

### 4.8. Analysis of the Contraction Capacity of a Collagen Matrix

Regarding HDF cells, 2.0 × 10^5^ were resuspended in 5x DMEM growth medium (Gibco, Waltham, MA, USA) in a 24-well plate, and a 2 mg/mL solution of collagen from bovine skin (Sigma–Aldrich, St. Louis, MO, USA) was added to each well. The pH of the solution was adjusted to 7.2. The plate was incubated at 37 °C for 45 min to allow solidification of the collagen gel. DMEM supplemented with 10% FBS and/or ML7 25 μM, as MYLK protein inhibitor, was added later. After 16 h, ML7 was cleared and ObHEx at the concentration of 0.002% in DMEM with 2% FBS were added. TGF-β 2.5 ng/mL was used as a positive control. The disk of collagen formed was immediately detached from the well by using a sterile micro spatula in order to promote its contraction. The areas of the disk of each treatment were measured at time 0 and 5 h and analyzed using ImageJ software. 

### 4.9. Analysis of the Degree of Actin Polymerization

Next, 1.5 × 10^5^ of HDF cells per well were grown in 24-well plate and the next day the medium was added with 2% FBS and the Cytochalasin B, which is an inhibitor of actin polymerization, at 2 μM for 30 min. After 30 min, on the cells, ObHEx (0.002% and 0.006%) was added together with TGF-β 2.5 ng/mL, used as a positive control, and ML7 25 µM, as a negative control of the MYLK enzyme. After 30 min of treatment, the cells were fixed with 4% paraformaldehyde (PFA) in buffer phosphate for 30 min on ice. The cells were washed with PBS and permeabilized with a solution of phosphate buffer and Triton-X100 at 0.2% for 30 min. Subsequently, the cells were incubated with a solution of 0.4 µM of phalloidin conjugated with rhodamine (Santa-Cruz Biotechnology, Dallas, TX, USA) for 1 h in the dark. At time 0 and after 18h, the fluorescence was measured at 540/570 nm using the Victor3 plate reader (PerkinElmer, Waltham, MA, USA). 

### 4.10. Analysis of SMAD2 Pathway

HDF cells were seeded at a density of 3 × 10^3^ in 96-well plates and after 16 h were transfected using X-tremeGene™ HP DNA transfection Reagent (Roche Diagnostics, Basel, Switzerland) with Smad2 reporter vector according to manufacturer’s instructions. After 24 h, the cells were treated with ObHEx or TGF-β 2.5 ng/mL, alone or in combination with ML7 25 μM for 24 h. At the end of the incubation, the cells were washed with PBS and the activity of luciferase was determined by using the SteadyGlo Luciferase assay system (Promega Corporation, Madison, WI, USA) in the Multiwell Plate Reader Victor Nivo (PerkinElmer, Waltham, MA, USA).

### 4.11. Analysis of Pro-Collagen I, Tropoelastin and Periostin Synthesis

Per well, 8 × 10^3^ HDF were grown in 96-well plates and treated with the 0.002% of ObHEx or TGFβ 2.5 ng/mL. After 24 h, the cells were processed for ELISA using monoclonal primary antibody anti-procollagen type I (sc-166572, Santa-Cruz Biotechnology, Dallas, TX, USA), followed by incubation with the secondary anti-mouse antibody labeled with peroxidase (170-6516, Biorad, Hercules, CA, USA). The supernatants of the cells were coated on another plate for the detection of tropoelastin and periostin using the anti-tropoelastin rabbit antibody (ab21600, Abcam, Cambridge, UK) or anti-periostin mouse antibody (sc-398631, Santa-Cruz Biotechnology, Dallas, TX, USA), followed by incubation with secondary antibody anti-rabbit labeled with peroxidase (170-6515, Biorad, Hercules, CA, USA). The colorimetric reaction was developed by adding 100 μL of an aqueous solution of OPD (O-phenylenediamine), 0.35 mg/mL in 50 mM citrate buffer, and 0.012% hydrogen peroxide (H_2_O_2_). After 30 min, the absorbance was measured at 490 nm using the multiplate reader Victor Nivo (PerkinElmer, Waltham, MA, USA).

### 4.12. Ex Vivo Tests

ImmunoHistoFluorescence (IHF) of MYLK and phosphorylated myosin was evaluated in skin explants of 44–47-year-old donors. Human skin explants were cut with punch biopsy curettes of 8 mm and cultured in 24-well plates in DMEM with 10% FBS and antibiotics. The obtained punches were treated with 0.002% and 0.006% of ObHEx for 24 h. They were incubated in 15% sucrose, then in 30% sucrose, and finally frozen. Next, 10 μm sections were obtained using the CM1520 cryostat (Leica Microsystems, Wetzlar, Germany). Slides with cryosections were hydrated for 30 min in PBS and placed in a “blocking” solution (6% BSA, 5% serum, 20 mM MgCl_2_, 0.2% Tween) for 1 h. Cryosections were incubated with the primary anti-MYLK rabbit antibody (1: 100, GeneTex, Irvine, CA, USA) and the antibody primary anti-phospho-myosin (1:100, LifeTechnologies, Carlsbad, CA, USA) for 16 h at 4 °C. The slides were washed with PBS for 30 min and then incubated with the secondary anti-rabbit Alexa-Fluor 546 antibody (1: 1000; A11035 ThermoFisher, Waltham, MA, USA) for 1 h. The nuclei were stained with DAPI (4‘, 6-5 diamidino-2-phenylindole) 1 μg/mL in PBS for 10 min. The images were acquired with a fluorescence microscope and analyzed with the ImageJ software. For IHF of tropoelastin and collagen I, skin explants of 36-year-old female donors were used. Skin biopsies were obtained as described above and pre-treated with 0.002% and 0.006% of ObHEx for 24 h. Stress with hydrocortisone at 10 ug/mL for 8 days was added in presence of ObHEx. Afterwards, biopsies were frozen as described above and the sections were incubated with the primary antibody anti-tropoelastin rabbit (1: 1000 ab21600, Abcam, Cambridge, UK) and anti-collagen I (1:100 C2456 Merck, Darmstadt, Germany) for 16 h at 4 °C. The slides were analyzed as described above and the images were acquired and analyzed with the ImageJ software.

### 4.13. Atomic Force Microscopy (AFM) in Skin Explants

Elasticity of skin samples untreated and treated with ObHEx was evaluated measuring their Young’s moduli by a method in nanometric scale based on Atomic Force Microscopy (AFM), developed not only for biological samples but also inorganic ones. These approaches are based on Hertz’s models’ assumptions and considered the sample and cantilever as two springs in a series in an AFM experiment setting. Briefly, skin explant treated with 0.006% of ObHEx and untreated skin samples were cut by cryostat in slices of 10 μm in thickness. Samples were stored at −12 °C, then washed with PBS and examined at a fixed temperature and relative humidity of 22 °C and 55%, respectively. Each sample was investigated by a NanoScope IIIA AFM in Force Spectroscopy Single Mode using a pyramidal tip (RESP-20) with a spring constant of 0.9 N/m. To calculate Young’s modulus, ten Force curves in ten different points of the same sample superficies were acquired. Each Force curve is a curve of deflection (Volt) versus displacement (nm) and can be converted in curves of Force (nN) versus separation (nm). In fact each *Force curve* reports a loading and an unloading curves. They represent the tip’s trend respect to the sample: when the tip is far from the sample, when they come into contact and the tip begins to deflect, and when it moves away again. Only the first part of these curves can be investigated. Each of these parts of the curves was fit with a standard Hertzian model, in particular, the typical Hertz equation for a pyramid tip, to extract the elastic modulus known as Young’s modulus in GPa. 

### 4.14. Statistical Analysis

All the values reported were the average of three independent experiments, each of which was performed in triplicate. The asterisks indicate statistical significance calculated in agreement with *t*-test: * means *p* value ≤ 0.05; ** *p* value ≤ 0.01; *** *p* value ≤ 0.001.

## Figures and Tables

**Table 1:** Molecular formula (MF), Retention time (RT), MS data of compounds identified in ObHEx by GNPS search library and literature study.

	Compound	MF (Mass Error ppm)	RT Min	Precursor Ions *m/z*	MS2 Ions *m/z* (Relative Intensity %)
**1**	Allantoin	C_4_H_6_N_4_O_3_ (2.5 ppm)	1.79	157.0360	114.0297 (87.36); 97.0031(100)
**2**	Pyridoxine	C_8_H_11_NO_3_ (2.4 ppm)	1.79	168.0660	150.0550 (100); 122.0599 (45.03)
**3**	Cyclic Adenosine Monophosphate	C_10_H_12_N_5_O_6_P (5.8 ppm)	1.88	328.0460	134.0461 (100)
**4**	Citric acid	C_6_H_8_O_7_ (4.2 ppm)	1.88	191.0194	111.0076 (52.28); 87.0075 (24.13)
**5**	L-tyrosine	C_9_H_11_NO_3_ (2.8 ppm)	2.73	180.0661	163.0392 (100); 119.0491 (68.65)
**6**	Arbutin	C_12_H_16_O_7_ (6.6 ppm)	2.95	271.0830	108.0205 (100); 71.0126 (5.30)
**7**	Adenosine	C_10_H_13_N_5_O_4_ (6.4 ppm)	3.08	266.0901	134.0461 (100)
**8**	Xanthosine	C_10_H_12_N_4_O_6_ (6.0 ppm)	3.39	283.0690	151.0252 (100)
**9**	L-phenylalanine	C_9_H_11_NO_2_ (3.0 ppm)	4.08	164.0711	147.0441 (100); 72.0078 (31.97)
**10**	Hydroxybenzoic acid glucoside	C_13_H_16_O_8_ (6.4 ppm)	4.43; 5.92	299.0780	179.0341 (2.00/23.36); 137.0233 (100/65.11)
**11**	Beta-D-fructofuranosyl 6-O-(4-hydroxybenzoyl)-alpha-D-glucopyranoside	C_19_H_26_O_13_ (4.8 ppm)	6.46	461.1312	137.0234 (100); 93.0333 (16.37)
**12**	Panthothenic acid	C_9_H_17_NO_5_ (4.6 ppm)	6.49	218.1033	146.0812 (75.65); 88.0391 (100)
**13**	Sinapyl alcohol diglucoside	C_23_H_34_O_14_ (3.8 ppm)	12.84	533.1885	209.0814 (98.19); 194.0578 (100)
**14**	Salvadoraside	C_34_H_48_O_18_ (4.4 ppm)	13.89; 14.11	743.2790	389.1612 (100)
**15**	Liriodendrin	C_34_H_46_O_18_ (4.4 ppm)	14.35; 14.53	741.2633	417.1556 (41.89); 181.0498 (100)
**16**	Syringaresinol glucoside	C_28_H_36_O_13_ (3.8 ppm)	15.73	579.2094	417.1560 (11.26); 181.0498 (100)
**17**	Hydroxydecanoate	C_10_H_20_O_3_ (4.3 ppm)	18.37; 24.43	187.1337	169.1229 (2.26/2.99); 141.1273 (6.73/100)
**18**	Myrianthic acid	C_30_H_48_O_6_ (4.4 ppm)	21.89	503.3389	485.3276 (51.41); 459.3505 (5.76)
**19**	Arjunolic acid and Asiatic acid	C_30_H_48_O_5_ (3.9 ppm)	24.13	487.3437	421.3104 (1.85); 409.3099 (5.57)
**20**	Dihydroxyoctadecenoic acid	C_18_H_34_O_4_ (6.1 ppm)	24.98; 25.61	313.2392	183.1383 (100); 129.0910 (67.67/56.97)
**21**	Glycerophosphocholine (18:3)	C_26_H_48_NO_7_P (4.3 ppm)	25.27	562.3163 (M+HCOOH−H)^−^	277.2172 (100); 224.0685 (0.97)
**22**	Glycerophosphoethanolamine (18:2)	C_23_H_44_NO_7_P (4.4 ppm)	26.71; 27.17	476.2793	279.2328 (100); 196.0374 (1.27/11.69)
**23**	Glycerophosphocholine (18:2)	C_26_H_50_NO_7_P (4.6 ppm)	26.79; 27.26	564.3322 (M+HCOOH−H)^−^	279.2328 (100); 224.0688 (0.86/10.27)
**24**	Glycerophosphoethanolamine (16:0)	C_21_H_44_NO_7_P (5.1 ppm)	27.68; 28.25	452.2795	255.2328 (100); 196.0373 (1.29/10.72)
**25**	Hederagenin	C_30_H_48_O_4_ (4.4 ppm)	28.77	471.3490	405.3163 (2.40); 393.3136 (6.12)
**26**	Hydroxyoctadecadienoic acid	C_18_H_32_O_3_ (5.1 ppm)	29.14; 29.53	295.2283	277.2172 (100); 171.1018 (64.06/58.66)
**27**	Glycerophosphoethanolamine (18:0)	C_23_H_48_NO_7_P (5.0 ppm)	30.87; 31.47	480.3109	283.2643(100); 196.0373 (1.53/11.63)
**28**	Coumaroyl triterpene	C_39_H_54_O_6_ (4.0 ppm)	32.85	617.3862	453.3399 (0.69); 145.0284 (54.98)
**29**	Gamma-Linolenic acid	C_18_H_30_O_2_ (5.4 ppm)	34.11	277.2177	233.2277 (0.71); 163.9689 (0.36)
**30**	Hydroxypalmitic acid	C_16_H_32_O_3_ (6.3 ppm)	36.28	271.2285	253.2173 (2.75); 225.2220 (100)

**Table 2:** Characteristics of the quantitative evaluation of lignanic and triterpenic compounds.

Compound	Range (nM)	Calibration Curve	R^2^	LOD (nM)	LOQ (nM)
Salvadoraside	250–25000	y = 1 × 10^7^ x	R^2^ = 1.0000	76	250
Liriodendrin	250–25000	y = 2 × 10^7^ x	R^2^ = 0.9995	76	250
Myrianthic acid	100–25000	y = 2 × 10^7^ x	R^2^ = 0.9999	8	25
Arjunolic acid	100–25000	y = 2 × 10^7^ x	R^2^ = 0.9971	3	10
Asiatic acid	100–25000	y = 3 × 10^7^ x	R^2^ = 0.9911	3	10
Hederagenin	100–25000	y = 4 × 10^7^ x	R^2^ = 0.9967	3	10

**Table 3:** Lignanic and triterpenic content.

Compound	Amount (µg/g of Extract)	% RSD
Salvadoraside	7.93	3.69
Liriodendrin	51.11	7.21
Myrianthic acid	22.09	3.85
Arjunolic acid	28.58	1.95
Asiatic acid	30.73	3.09
Hederagenin	78.21	0.61

## Data Availability

Data is contained within the article.
